# Development and pilot test of ComCare – a questionnaire for quick assessment of communicative and social competences in medical students after interviews with simulated patients

**DOI:** 10.3205/zma001464

**Published:** 2021-03-15

**Authors:** Julia Gärtner, Sarah Prediger, Sigrid Harendza

**Affiliations:** 1 University Medical Center Hamburg-Eppendorf, Department of Internal Medicine, Hamburg, Germany; 2 University Medical Center Hamburg-Eppendorf, III. Medical Clinic, Hamburg, Germany

**Keywords:** assessment, communication, interpersonal skills, social competence, medical students, undergraduate medical education

## Abstract

**Background: **Physicians’ communicative and social competences are highly relevant for doctor-patient relationships. Simulation-based learning is frequently used to provide students with learning experiences resembling realistic medical situations. This study aims to assess communication and interpersonal skills in medical students after simulated consultations with a newly designed short questionnaire.

**Methods:** In 2019, 103 final year students participated in a simulated consultation hour seeing four simulated patients. Communicative and social competences were assessed by a questionnaire including items for communication (Com) and interpersonal (Care) skills. The questionnaire was used by the simulated patients (ComCareP) after each consultation and as self-assessment by the students (ComCareD) after the fourth consultation. An explorative factor analysis was performed and the results of ComCareP and ComCareD were compared with respect to students’ sex and advancement in their final year.

**Results: **All ComCareP items loaded on one factor, which explained 50.7% of the variance. The participants self-assessed their communication and interpersonal skills significantly better than the simulated patients. No significant differences were found for students’ sexes or advancement in their final year except for the item “responding to patients’ needs satisfactorily” which was significantly lower in students at the end of their final year. Patients’ general “satisfaction with the consultation” was higher while physicians’ general “satisfaction with the consultation” was lower than their total ComCare mean score. The general satisfaction with the consultation showed a significant positive correlation with both ComCares’ total mean scores.

**Conclusion: **The ComCare measures communication and interpersonal skills as one factor. It can be used directly after consultations and shows significant positive correlation with the general satisfaction with a consultation. Since simulated patients’ satisfaction with the consultation was higher than their ComCare score, other factors than communication and interpersonal skills could play a role for patient satisfaction with a conversation and need to be further investigated.

## Zusammenfassung

**Hintergrund: **Die kommunikativen und sozialen Kompetenzen Mediziner*innen sind für die Beziehung von Ärzt*innen und Patient*innen von großer Relevanz. Simulationsbasiertes Lernen wird häufig eingesetzt, um den Studierenden Lernerfahrungen zu vermitteln, die realistischen medizinischen Situationen ähneln. Diese Studie zielt darauf ab, die kommunikativen und sozialen Kompetenzen von Medizinstudierenden im Anschluss an simulierte Konsultationen mit einem neu gestalteten kurzen Fragebogen zu beurteilen.

**Methoden:** Im Jahr 2019 nahmen 103 Studierende während des Praktischen Jahres (PJ) an einer simulierten Sprechstunde mit vier simulierten Patient*innen teil. Die kommunikativen und sozialen Kompetenzen wurden anhand eines Fragebogens bewertet, welcher Items für Kommunikations- (Com) und zwischenmenschliche (Care) Fähigkeiten enthielt. Der Fragebogen wurde von den simulierten Patient*innen (ComCareP) nach jeder Konsultation und als Selbsteinschätzung der Studierenden (ComCareD) nach der vierten Konsultation verwendet. Es wurde eine explorative Faktorenanalyse durchgeführt und die Ergebnisse von ComCareP und ComCareD wurden im Hinblick auf das Geschlecht und den Fortschritt der Studierenden im PJ verglichen.

**Ergebnisse: **Alle ComCareP-Items luden auf einen Faktor, was 50,7% der Varianz erklärte. Die Teilnehmer*innen bewerteten ihre Kommunikations- und zwischenmenschlichen Fähigkeiten signifikant besser als die simulierten Patient*innen. Es wurden keine signifikanten Unterschiede in Bezug auf das Geschlecht oder den Fortschritt der Studierenden im PJ gefunden, mit Ausnahme des Items "zufriedenstellend auf die Bedürfnisse der Patient*innen eingehen", das bei den Studierenden am Ende des PJs signifikant niedriger war. Die allgemeine „Zufriedenheit der Patient*innen mit dem Erstgespräch“ war höher, während die allgemeine „Zufriedenheit der Ärzt*innen mit dem Erstgespräch“ niedriger war als der Gesamtmittelwert von ComCare. Die allgemeine „Zufriedenheit mit dem Erstgespräch“ zeigte eine signifikant positive Korrelation mit den Gesamtmittelwerten beider ComCare Bögen.

**Schlussfolgerung: **Der ComCare misst Kommunikation und zwischenmenschliche Fähigkeiten als einen Faktor. Er kann direkt nach einer Konsultation eingesetzt werden und zeigt eine signifikant positive Korrelation mit der allgemeinen Zufriedenheit mit einer Beratung. Da die Zufriedenheit der simulierten Patient*innen mit dem Erstgespräch höher war als deren ComCare-Gesamtwert, könnten neben der Kommunikation und den zwischenmenschlichen Fähigkeiten noch andere Faktoren eine Rolle für die Zufriedenheit der Patient*innen mit dem Erstgespräch spielen und müssen weiter untersucht werden.

## Introduction

Physicians’ communicative and social competences play an important role for patients’ acceptance of disease and adherence to therapy [1], [2]. They also enable physicians to establish a therapeutic relationship with the patients [3]. Communicative and social competences including the ability to connect with patients are critically important for patients’ satisfaction with a consultation [4], [5]. Additionally, they seem to be gender-related with female physicians showing a more patient-centered communication style than male physicians [6], [7]. Competences generally include the capability to use knowledge, skills, and attitudes to perform tasks [8] and are integrated in the professional repertoire of an individual [9]. The importance of improving physicians’ and medical students’ professional attitudes and interpersonal skills during undergraduate and postgraduate education had already been emphasized 30 years ago [10]. Communicative skills can be understood as a behavioral aspect of interpersonal skills [11] and help medical students to acquire competence in relationship building [12]. Communication and interpersonal skills are recommended to become part of the core set of clinical skills for undergraduate and postgraduate medical training [13]. They are already included in the National Competence Based Catalogue of Learning Objectives for Undergraduate Medical Education (NKLM) [14] and other international competence frameworks, e.g. the Dutch Blueprint [15] or the Swiss Catalogue of Learning Objectives [https://www.scienceopen.com/document?vid=fe14d640-5778-46e4-a702-723aefc5e2bb]. However, the actual implementation in the curriculum is only partially realized [16] and will need further improvement [17].

The Basel Consensus Statement “Communicative and Social Competencies in Medical Education” describes communication and interpersonal competences that medical students should have achieved at the end of their undergraduate studies [18]. Additionally, the skills related with these competences remain an important part of physicians’ lifelong learning [19] and are central for providing empathetic patient care [9], [12]. During undergraduate medical education a decline of empathy in medical students has been described [20], [21]. Another study found that empathy can be preserved despite a previous decline [22]. However, various studies show that specific training programs can enhance communication and interpersonal skills in medical students [23], [24], [25], [26]. Courses with theoretical content about communicative and social competence combined with practical exercises e.g. role-playing scenarios with peers and simulated patients [27], provide an effective opportunity to learn and exercise communication and interpersonal skills [23], [28], [29]. Furthermore, simulations – as an experience of realistic situations in a safe environment – provide the possibility for medical students to self-responsibly transfer knowledge into practice [30], [31]. This is also highly valued by the students [32]. 

An important threshold where medical students transfer their theoretical knowledge into practice is the final year. However, final year students tend to overestimate their own performance [33] and still show deficits in their communicative skills, e.g. during physical examinations [34]]. It has been shown that medical students’ level of communication skills increases during final year [35]. At the same time, this final phase of undergraduate training contains challenges which can lead to an increased sense of stress [36] and uncertainty [37]. In postgraduate training, these indicators of difficulties correlate negatively with residents’ communicative and interpersonal skills [38], [39]. Based on a previously developed assessment [40], [41], simulating a first day of residency for final year medical students in the physician’s role, we developed a shortened training of this competence-based format with a focus on history taking, patient documentation, and case presentation [42]. This training includes a simulated consultation hour for the medical students to exercise their communication and interpersonal skills with simulated patients and a short questionnaire was required for quick assessment of students’ skills between the patient interviews. The aim of this study was to investigate final-year students’ communicative and interpersonal skills during a competence-based training including ratings from the simulated patients’ perspectives and students’ self-ratings to provide quick formative feedback. We compared students’ rating results by their sex and phase of the final year.

## Methods

### Procedure

The study took place at the University Center Hamburg-Eppendorf in October and December 2019. Evaluating communication and interpersonal skills in medical students was part of an assessment center simulating physicians’ tasks during a first day of residency. This simulation was based on a validated 360-degree competence-based assessment procedure [40], [41] with final year medical students in the physician’s role. It included a consultation hour with four simulated patients per participant, a management phase where patient documentation took place and diagnostic tests could be electronically ordered, and the presentation of one patient case in a handover situation based on information from the patient’s history, physical examination, and test results. The participants discussed further management of the presented cases under supervision of a senior physician. The simulation was framed by a briefing and debriefing phase. Communication and interpersonal skills were assessed by the simulated patients after every interview and as self-assessment by the participants after the fourth interview. The patient cases were based on real patients and designed in a way that they required analytical thinking [40]. Furthermore, every simulated patient’s role included specific personality characteristics (e.g. becoming angry very easily, being very talkative).

#### Participants

In total, 103 medical students (female: *n*=65, male: *n*=38) from the Medical Faculty of Hamburg University participated in the simulation in their final year of a 6-year undergraduate medical program. Participation was voluntary and included informed and written consent. All participants received a certificate of attendance after the debriefing. All data were anonymized for analysis. This study was approved by the Ethics Committee of the Chamber of Physicians, Hamburg (reference number: PV3649). Data from one participant had to be excluded from the analysis due to an incomplete data set. Data from 102 participants were included in the analysis (n=53 from final year students in their first four months, *n*=17 from final year students in their second four months, and n=32 from final year students in their third four months). 

#### Instrument

Existing instruments for measuring communicative and interpersonal competences are often very long and either only include scales that relate to the evaluation of patients’ perspective (real or simulated), to specific contexts of care, or to specifically investigate medical students’ communication competences during medical training [43]. Furthermore, rating scales for communicative and interpersonal competences are often applied within the common OSCE format [43], [44], [45], [46], [47]. To assess communication and interpersonal competences after the physician-patient encounters in our simulation we developed a short eight-item questionnaire (ComCare) (see attachment 1 and attachment 2 ) consisting of three items related to communication skills, four items related to interpersonal skills, and one general item assessing the satisfaction with the consultation. This questionnaire was specifically fitted to the requirements of our training format:

1. the need for quick evaluation by the simulated patients directly after the consultations and
2. measuring these skills within a complex setting without participants being aware that these skills were assessed for feedback purposes.

The participants were not aware of the particular items of the questionnaire before the simulation to foster natural communication and interpersonal behavior and to avoid item specific behavior. Communication and interpersonal skills have been proposed in a framework to be inseparably connected [11], [12]. Therefore, we constructed our new ComCare questionnaire from questionnaires used in previous projects for either measuring communication or interpersonal skills after simulated patient consultations. The communication items of ComCare were based on a questionnaire by Bittner et al [48] that was used to assess undergraduate medical students’ communication skills after consultations with simulated patients via skype. The items related to interpersonal skills were derived from the Consultation and Relational Empathy (CARE) questionnaire [49] that was used by the simulated patients in our previous competence-based assessments to evaluate medical students’ social competence [40], [50]. The combination of very few items from these two questionnaires fitted both our purposes. 

We created two versions of the ComCare questionnaire. One version (ComCareP) was used by the simulated patients after every interview. Based on this questionnaire we designed a self-assessment version (ComCareD) which was filled out by the participating students in their role as physicians (“doctor”) after the fourth patient interview. Communication related items of ComCare included “use of understandable language”, “satisfactorily responding to the patient’s needs” and “comprehensibly explaining the next steps of diagnostics and treatment”. CARE derived items comprised “attentive listening”, “showing sincere interest”, ”being compassionate”, and “creating a comfortable atmosphere”. The eighth item was a general statement about “satisfaction with the consultation”. All items had to be assessed on a five-point Likert scale (1=full disagreement to 5=full agreement). While in ComCareP all items were phrased from the patient’s perspective, e.g. “The doctor used language I could understand”, all questions in ComCareD were designed from the physician’s perspective, e.g. “I used language the patient could understand”.

#### Statistical analysis

For statistical analysis, means and standard deviations were calculated for all assessed items of communication and interpersonal skills (ComCare) using SPSS Statistics 26. The structure of the newly designed questionnaire was examined with an explorative factor analysis of its seven communication and interpersonal skills related items. Cronbach’s α was calculated for the questionnaire. To examine differences between the sexes, we used an independent samples t-test. To analyse differences between the three student groups with respect to their advancement in the final year (first, second, and third four months) we conducted an analysis of variance (ANOVA) and a Bonferroni post-hoc test. Additionally, Cohen’s d was calculated for effect sizes. To examine relationships between the general item “satisfaction with the consultation” and the seven ComCare items, correlations were calculated (Pearson’s *r*).

## Results

Factor analysis of ComCareP revealed one factor (KMO=.82), which explains 51.2% of variance. Individual item loadings are shown in table 1 (Tab. 1) and Cronbach’s α was .84. The total score mean for participants’ assessment of communicative and interpersonal skills with the ComCareP was 2.79±.38 (see table 2 (Tab. 2)), with the highest value for the item ”The physician showed sincere interest in me as a human being”, 3.43±.59, and the lowest value for the item “The physician used language I could understand”, 2.31±.30. All item means as well as the total mean score of the ComCareD (4.16±.34) were significantly (p<.001) higher than all item means and the total mean score of the ComCareP. A significantly (p<.001) lower rating for general “satisfaction with the consultation” was found in ComCareP (3.20±.52) versus ComCareD (3.59±.71. Ratings in ComCareP were also significantly lower for all items compared to ComCareD in female and male participants and in all three thirds of advancement in students’ final year. No significant differences were found between female and male participants for any item of ComCareP and ComCareD.

Comparing participants according to their advancement in the final year (see table 3 (Tab. 3)) no significant differences were found between the three groups except for the mean score for the ComCareP item “responding to the patient’s needs satisfactorily”. The mean for this item was significantly higher (p<.01) for students in the second four months of their final year (3.23±.53) than for students in the third four months of their final year (2.75±.47) with a large effect (Cohen’s d=.977). For all participants, regardless of their sex or their advancement in the final year, the ComCareP’s mean for general “satisfaction with the consultation” was higher than the total mean score for the ComCare items while ComCareD showed inverted results. 

ComCareP items showed medium to strong positive correlations and ComCareD items showed low to medium positive correlations with the general “satisfaction with the consultation” (see table 4 (Tab. 4)). In ComCareP, the strongest significant positive correlation (r=.650, p<.001) was found with the item “creating a comfortable atmosphere”, followed by “responding to the patient’s needs satisfactorily” (r=.621, p<.001), and “showing sincere interest” (r=.633, p<.001). In ComCareD, the strongest significant positive correlation (r=.428, p<.001) was identified with the item “comprehensibly explaining the next steps of diagnostics and treatment”. The general satisfaction with the consultation showed a significantly strong positive correlation with ComCareP’s total score mean (r=.765, p<.001) and a significantly medium positive correlation (r=.486, p<.001) with ComCareD’s total score.

## Discussion

Our newly designed ComCare questionnaire for the assessment of communication and interpersonal skills revealed one factor in the explorative factor analysis. This underscores the underlying framework which postulates that communication and interpersonal skills are inseparably connected [11], [12]. In communicating with real patients, physicians’ lack of interpersonal skills has been shown to be an obstacle to successful conversations [51]. Medical schools are increasingly providing longitudinal communication curricula as suggested by the NKLM [http://www.nklm.de, retrieved 29.3.2020] in undergraduate and postgraduate learning, where basic communication techniques are studied earlier [52], [53] and communication trainings, which progressively require interpersonal skills, are scheduled later [53], [54]. To emphasize that communication and interpersonal skills are connected, the term “interactional skills” is being used and recommendations for future research in this field have been given [55]. With the ComCare questionnaire we provide an instrument for assessment and feedback that could be used in trainings for challenging or difficult conversations [56] where communication and interpersonal skills are highly required [57]. Its great advantage over existing instruments is its quick use during simulations after every conversation, which allows for fast formative feedback to simulation participants.

With respect to communication and interpersonal skills, female medical students showed slightly better scores in the United States medical licensing examination step 2 than male examinees [58]. In our study, the comparison of gender groups for ComCareP and ComCareD revealed no significant differences. This finding is somewhat difficult to interpret but requires special attention for further studies with the ComCare instruments, because very few communication assessment instruments in medical education have been found to include an appropriate focus on gender [59]. Regarding the students’ advancement in their final year we only found one significant difference in the ComCareP’s score for the item “responding to the patient’s needs satisfactorily” with a lower mean achieved by students in the third four months of the final year compared to students in the second four months of the final year. In a patient study, the item “sensitivity for patients’ needs” most highly correlated with overall patient satisfaction [60]. Overall satisfaction with the consultation in the ComCareP for the two student groups was not significantly different and in general “creating a comfortable atmosphere” most highly correlated with overall satisfaction. Even though it has been described that communication and interpersonal skills can decrease during undergraduate education [61] this finding in just one item of an instrument that measures a single factor does not appear to be of major relevance.

Interestingly, students’ total ComCareD score was significantly higher than the simulated patients’ total ComCareP score, but their ComCareD score for general “satisfaction with the consultation” was lower than their total score, while simulated patients’ ComCareP score for general “satisfaction with the consultation” was higher than their total ComCareP score. A study amongst patients and physicians even found that patients’ overall satisfaction was even higher than physicians’ satisfaction [62]. Similarly, physicians were found to view the overall benefits patients gained from their consultation more negatively than the patients themselves [63]. Since in our study the correlation between general “satisfaction with the consultation” and total mean score was stronger in ComCareP than in ComCareD, the individually assessed communication and interpersonal skills seem to be more relevant for patients’ general “satisfaction with the consultation” than for the students’ general “satisfaction with the consultation”. The highest correlations of ComCareP items with the general “satisfaction of the consultation” were “creating a comfortable atmosphere”, “showing sincere interest”, and “satisfactorily responding to the patient’s needs”. Data from physician-rating websites that highly correlated with patients’ overall satisfaction were aspects related to the factors atmosphere, interest, and patients’ needs, namely physicians’ friendly manner, attentive listening, and handling concerns in an empathetic way [64]. According to our findings with ComCareD, medical students seem to have a completely different perspective on communication and interpersonal skills and general satisfaction with a patient encounter. They self-assess their skills with high values, suggesting that their perspective could be focused on communication techniques they learned in a course [28] or that were assessed with a checklist in an objective structured clinical examination (OSCE) [65]. The highest correlation with students’ general satisfaction in ComCareD was with the item “comprehensibly explaining the next steps of diagnostics and treatment”. In the debriefing of our assessment students report a lack of medical knowledge during the consulting hour (data not shown) and deficits in clinical reasoning skills during history taking have been identified [66]. Recognizing these deficits which could have led to lower scores for the item “comprehensibly explaining the next steps of diagnostics and treatment” might be a reason for the low scores in general satisfaction because health care and medical students grow up in a culture where they ‘learn’ to hide their personal deficits in knowledge [67]. This can be possible in courses and OSCEs, because students learn to develop strategies to appear certain and competent [68], but it is impossible in a physician-patient encounter if one feels responsible for best possible care. This finding could be a hint that communication and interpersonal skills need to be taught in a longitudinal curriculum with increasingly difficult medical content to enable the students to exercise these skills while remaining focused the differential diagnosis of a medical problem at the same time.

A strength of our study is the high number of students who participated in our assessment. Furthermore, the construction of our ComCare questionnaire was based on operationalized criteria and showed a good internal consistency of .84. A weakness of our study is that the students were unevenly distributed in the three four-month periods of the final year and that they were included in this study on a first-come, first served basis, which could have led to a self-selection of very motivated students. We did not find any differences between the three groups which might be due to the weakness that the groups were not studied longitudinally but resembled a cross sectional convenience sample. Another weakness is the lack of a validation study of our new short instrument preceding our assessment. Furthermore, the ComCare items were not anchored by examples to ensure a quick read during the few minutes between the consultations. However, all simulated patients received a training with the ComCareP before the assessment to standardize their answers watching simulated physician-patient conversations. Despite the limitations, our findings with the ComCareP suggest that even though general satisfaction with a physician-patient encounter by simulated patients is associated with operationalized items, satisfaction is higher than the mean score of all communication and interpersonal skills items of the questionnaire, suggesting, that other factors, e.g. physicians’ personality, could play a role for feeling satisfied. Further studies need to explore additional factors besides communication and interpersonal skills leading to patients’ general satisfaction with physician-patient encounters.

## Conclusion

With the ComCare questionnaire communication and interpersonal skill can be robustly and quickly assessed. The differences in total ComCare scores versus the general satisfaction with the consultation in simulated patients’ assessment and students’ self-assessment suggest that additional aspects could play a role in expressing communicative and social competences. Further studies for the development and validation of ComCare and investigations of other factors of influence on communicative and social competences should be performed.

## Funding

This project was supported by the Joachim Herz Foundation, the Medical Faculty of Hamburg University, and the University Hospital Hamburg-Eppendorf.

## Profiles

**Location’s name: **University of Hamburg

**Field of study/profession:** Medicine

**Number of learners per year or semester: **Approx. 360 students per year

**Is a longitudinal communication curriculum implemented? **The model study program iMED contains a longitudinal KUMplusKOM curriculum, which includes communication and practical skills.

**In which semesters are communication and social skills taught?** Starting with the 1^st^ semester and in all semesters, which are divided into modules in iMED.

**What teaching formats are used?** Lecture, seminar, role-play, simulated patients

**In which semesters are communicative and social skills assessed (formative or relevant for passing and/or graded)? **In all module examinations that include an OSCE (from the 3^rd^ semester), relevant for passing 

**Which examination formats are used? **OSCE

**Who (e.g., clinic, institution) is in charge of development and implementation? **There is an overarching KUMplusKOM planning group headed by the directors of Medical Psychology and General Medicine. Staff members from diverse disciplines work together. The implementation of the concepts takes place in the disciplines with communicative learning objectives.

## Current professional roles of the authors

- Julia Gärtner, M.A., is a sociologist and scientific research associate in the section “Educational Research”, III. Department of Internal Medicine, University Hospital Hamburg-Eppendorf, Germany
- Sarah Prediger, M.A., is a sociologist and scientific research associate in the section “Educational Research”, III. Department of Internal Medicine, University Hospital Hamburg-Eppendorf, Germany
- Sigrid Harendza, MME, full professor of internal medicine/educational research, is heading the section “Educational Research”, III. Department of Internal Medicine, University Hospital Hamburg-Eppendorf. She was vice-dean of education from 2006-2007 at the Medical Faculty of Hamburg University, Germany, and received the Ars legendi award for excellent academic teaching in 2006.

## Competing interests

The author declares that she has no competing interests.

## Supplementary Material

ComCareP

ComCareD

## Figures and Tables

**Table 1:**
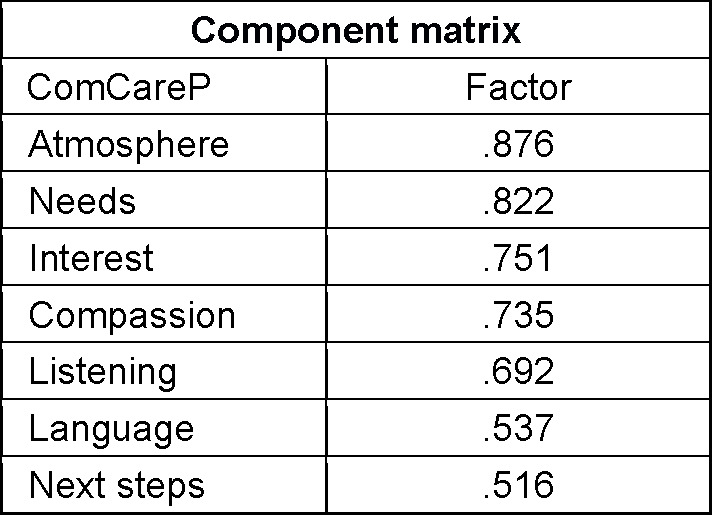
Factor analysis

**Table 2:**
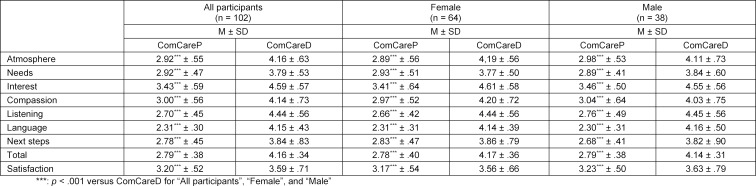
Means of ComCare questionnaires and individual ComCare items of all participants and by sex

**Table 3:**
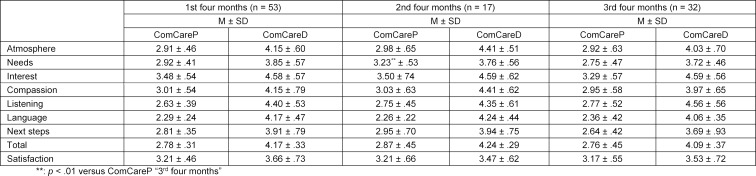
Means of ComCare questionnaires and individual ComCare items by groups of study progress in the final year

**Table 4:**
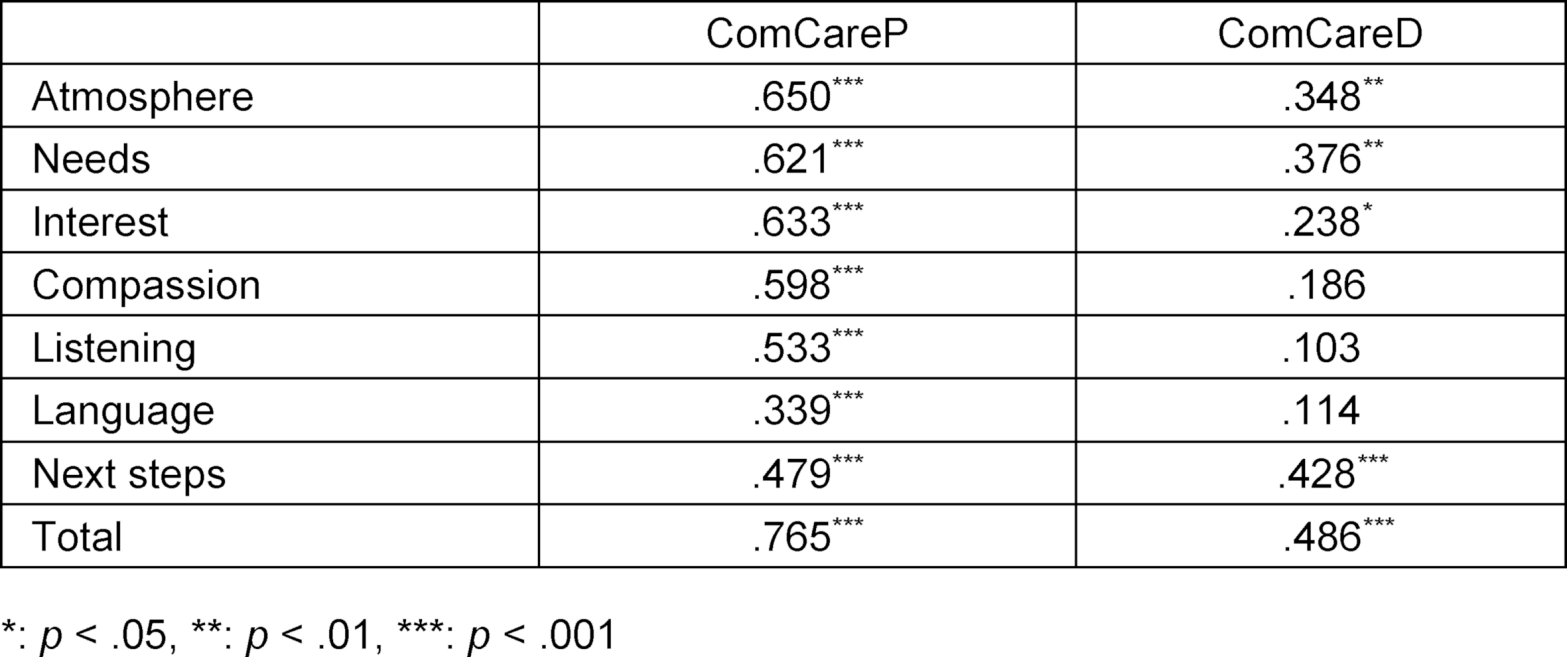
Correlation of Satisfaction with all ComCare items

## References

[1] Zolnierek KB, Dimatteo MR (2009). Physician communication and patient adherence to treatment: a meta-analysis. Med Care.

[2] Bryl N, Horst-Sikorska W, Ignaszak-Szczepaniak M, Marcinkowska M, Michalak M, Sewerynek E (2012). Influence of social competence of physicians on patient compliance with osteoporosis medications - a study on Polish postmenopausal women. Ginekol Pol.

[3] Loignon C, Haggerty JL, Fortin M, Bedos CP, Allen D, Barbeau D (2010). Physicians' social competence in the provision of care to persons living in poverty: research protocol. BMC Health Serv Res.

[4] DiMatteo MR (1979). A social-psychological analysis of physician-patient-rapport: toward a science of the art of medicine. J Soc Issues.

[5] Janssen SM, Lagro-Janssen AL (2012). Physician's gender. Communication style, patient preferences and patient satisfaction in gynecology and obstetrics: a systematic review. Patient Educ Couns.

[6] Roter DL, Hall JA (2004). Physician gender and patient-centered communication: A critical review of empirical research. Annu Rev Public Health.

[7] Hall JA, Roter DL (1998). Medical communication and gender: a summary of research. JGSM.

[8] Hager P, Gonczi A (1996). What is competence?. Med Teach.

[9] Duffy FD, Gordon GH, Whelan G, Cole-Kelly K, Frankel R (2004). Assessing Competence in communication and interpersonal skills: The Kalamazoo II report. Acad Med.

[10] McCue JD (1985). Influence of medical and premedical education on important personal qualities of physicians. Am J Med.

[11] Kanning UP (2002). Soziale Kompetenz - Definition, Strukturen und Prozesse. Z Psychol.

[12] Dyche, L (2007). Interpersonal skill in medicine: the essential partner of verbal communication. J Gen Intern Med.

[13] Magen E, DeLisser HM (2017). Best practices in relational skills training for medical trainees and providers: an essential element of addressing adverse childhood experiences and promoting resilience. Acad Pediatr.

[14] Fischer MR, Bauer D, Mohn K (2015). Finally finished! National competence based catalogues of learning objectives for undergraduate medical education (NKLM) and dental education (NKLZ) ready for trial. GMS Z Med Ausbild.

[15] Metz JC, Verbeek-Weel AM, Huisjes HJ (2001). Blueprint 2001: training of doctors in The Netherlands. Adjusted objectives of undergraduate medical education.

[16] Steinhäuser J, Chenot JF, Roos M, Ledig T, Joos S (2013). Competence-based curriculum development for general practice in Germany: a stepwise peer-based approach instead of reinventing the wheel. BMC Res Notes.

[17] Hawkins RE, Welcher CM, Holmboe ES, Kirk LM, Norcini JJ, Simons KB, Skochelak SE (2015). Implementation of competency-based medical education: are we addressing the concerns and challenges?. Med Educ.

[18] Kiessling C, Dieterich A, Fabry G, Hölzer H, Langewitz W, Mühlinghaus I, Pruskil S, Scheffer S, Schubert S, Committee Communication and Social Competencies oft he Association for Medical Education / Gesellschaft für Medizinische Ausbildung, Basel Workshop Participants (2010). Communication and social competences in medical education in German-speaking countries: the Basel consensus statement: results of a Delphi survey. Patient Educ Couns.

[19] Leach DC (2002). Competence is a habit. JAMA.

[20] Hojat M, Vergare MJ, Maxwell K, Brainard G, Herrine SK, Isenberg GA, Veloski J, Gonnella JS (2009). The devil is in the third year: a longitudinal study of erosion of empathy in medical school. Acad Med.

[21] Chen DC, Kirshenbaum DS, Yan J, Kirshenbaum E, Aseltine RH (2012). Characterizing changes in student empathy throughout medical school. Med Teach.

[22] Hegazi I, Hennessy A, Wilson I, Kondo M (2017). Empathy levels in medical students: do they really change over time?. Empathy - an evidence-based interdisciplinary perspective.

[23] Hausberg MC, Hergert A, Kröger C, Bullinger M, Rose M, Andreas S (2012). Enhancing medical students' communication skills: development and evaluation of an undergraduate training program. BMC Med Educ.

[24] Shankar PR, Dubey AK, Balasubramanium R, Dwivedi NR (2013). Students attitude towards communication skills learning in a Caribbean medical school. Australas Med J.

[25] Pruskil S, Deis N, Druener S, Kiessling C, Philipp S, Rockenbauch K (2015). Implementation of "social and communicative competences" in medical education. The importance of curriculum, organisational and human resource development. GMS Z Med Ausbild.

[26] Simmeroth-Nayda A, Weiss C, Fischer T, Himmel W (2012). Do communication training programs improve students' communication skills? - a follow-up study. BMC Res Notes.

[27] Nestel D, Tierney T (2007). Role-play for medical students learning about communication: guidelines for maximizing benefits. BMC Med Educ.

[28] Bachmann C, Barzel A, Roschlaub S, Ehrhardt M, Scherer M (2013). Can a brief two-hour interdisciplinary communication skills training be successful in undergraduate medical education?. Patient Educ Couns.

[29] Fernández-Olano C, Montoya-Fernández J, Salinas-Sanchez AS (2008). Impact of clinical interview training on the empathy level of medical students and medical residents. Med Teach.

[30] Nestel D, Groom J, Eikeland-Husebø S, O'Donnell JM (2011). Simulation for learning and teaching procedural skills: the state of the science. Simul Healthc.

[31] Weller JM, Nestel D, Marshall SD, Brooks PM, Conn JJ (2012). Simulation in clinical teaching and learning. Med J Aust.

[32] Weller JM (2004). Simulation in undergraduate medical education: bridging the gap between theory and practice. Med Educ.

[33] Störmann S, Stankiewicz M, Raes P, Berchtold C, Kosanke Y, Illes G, Loose P, Angstwurm MW (2016). How well do final year undergraduate medical students master practical clinical skills?. GMS J Med Educ.

[34] Krautter M, Diefenbacher K, Koehl-Hackert N, Buss B, Nagelmann L, Herzog W, Jünger J, Nikendei C (2015). Short communication: Final year students' deficits in physical examination skills performance in Germany. Z Evid Fortbild Qual Gesundhwes.

[35] Gude T, Vaglum P, Anvik T, Bearheim A, Fasmer OB, Grimstad H, Hjortdal P, Holen A, Nordoy T, Eide H (2009). Do physicians improve their communication skills between finishing medical school and completing internship? A nationwide prospective observational cohort study. Patient Educ Couns.

[36] Kumar B, Shah MAA, Kumari R, Kumar A, Kumar J, Tahir A (2019). Depression, anxiety, and stress among final-year medical students. Cureus.

[37] Schrauth M, Weyrich P, Kraus B, Jünger J, Zipfel S, Nikendei C (2009). Workplace learning for final-year medical students: a comprehensive analysis of student's expectancies and experiences. Z Evid Fortbild Qual Gesundhwes.

[38] Pereira-Lima K, Loureiro SR (2015). Burnout, anxiety, depression, and social skills in medical residents. Psychol Health Med.

[39] Tejwani V, Ha D, Isada C (2016). Observations: Public speaking anxiety in graduate medical education - - a matter of interpersonal and communication skills?. J Grad Med Educ.

[40] Prediger S, Schick K, Fincke F, Fürstenberg S, Oubaid V, Kadmon M, Berberat PO, Harendza S (2020). Validation of a competence-based assessment of medical students' performance in the physician's role. BMC Med Educ.

[41] Harendza S, Berberat PO, Kadmon M (2017). Assessing competences in medical students with a newly designed 360-degree examination of a simulated first day of residency: a feasibility study. J Community Med Health Educ.

[42] Harendza S, Gärtner J, Zelesniack E, Prediger S (2020). Evaluation of a telemedicine-based training for final-year medical students including simulated patient consultations, documentation and case presentation. GMS J Med Educ.

[43] Schirmer JM, Mauksch L, Lang F, Marvel MK, Zoppi K, Epstein RM, Brock D, Pryzbylski M (2005). Assessing communication competence: a review of current tools. Fam Med.

[44] Hodges B, McIlroy JH (2003). Analytic global OSCE ratings are sensitive to level of training. Med Educ.

[45] Scheffer S, Muehlinghaus I, Froehmel A, Ortwein H (2008). Assessing students' communication skills: validation of a global rating. Adv Health Sci Educ Theory Pract.

[46] Setyonugroho W, Kennedy KM, Kropmans TJ (2015). Reliability and validity of OSCE checklists used to assess the communication skills of undergraduate medical students: a systematic review. Patient Educ Couns.

[47] Cömert M, Zill JM, Christalle E, Dirmaier J, Härter M, Scholl I (2016). Assessing communication skills of medical students in objective structured clinical examinations (OSCE) - - a systematic review of rating scales. PLoS One.

[48] Bittner A, Bittner J, Jonietz A, Dybowski C, Harendza S (2016). Translating medical documents improves students' communication skills in simulated physician-patient encounters. BMC Med Educ.

[49] Mercer SW, Maxwell M, Heaney D, Watt GC (2004). The consultation and relational empathy (CARE) measure: development and preliminary validation and reliability of an empathy-based consultation process measure. Fam Pract.

[50] Wijnen-Meijer M, Van der Schaaf M, Booji E, Harendza S, Boscardin C, Van Wijngaarden, Ten Cate TJ (2013). An argument-based approach to the validation of UHTRUST: can we measure how recent graduates can be trusted with unfamiliar tasks?. Adv Health Sci Educ Theory Pract.

[51] Branson CF, Houseworth J, Chipman JG (2019). Communication deficits among surgical residents during difficult patient family conversations. J Surg Educ.

[52] Keifenheim KE, Teufel M, Ip J, Speiser N, Leehr EJ, Zipfel S, Herrmann-Werner A (2015). Teaching history taking to medical students: a systematic review. BMC Med Educ.

[53] Vermylen JH, Wayne DB, Cohen ER, McGaghie WC, Wood GJ (2020). Promoting readiness for residency: embedding simulation-based mastery learning for breaking bad news into the medicine sub-internship. Acad Med.

[54] Van Weel-Baumgarten EM, Brouwers M, Grosfeld F, Jongen Hermus F, Van Dalen J, Bonke B (2012). Teaching and training in breaking bad news at the Dutch medical schools: a comparison. Med Teach.

[55] Sanson-Fisher R, Hobden B, Carey M, Mackenzie L, Hyde L, Shepherd J (2019). Interactional skills training in undergraduate medical education: ten principles for guiding future research. BMC Med Educ.

[56] Haglund MM, Rudd M, Nagler A, Prose NS (2015). Difficult conversations: a national course for neurosurgery residents in physician-patient communication. J Surg Educ.

[57] Adams J, Murray R (1998). The general approach to the difficult patient. Med Clin North Am.

[58] Cuddy MM, Swygert KA, Swanson DB, Jobe AC (2011). A multilevel analysis of examinee gender, standardized patient gender, and United States medical licensing examination step 2 clinical skills communication and interpersonal skills scores. Acad Med.

[59] Dielissen P, Bottema B, Verdonk P, Lagro-Janssen T (2011). Attention to gender in communication skills assessment instruments in medical education: a review. Med Educ.

[60] Clark PA (2003). Medical practices' sensitivity to patients' needs. Opportunities and practices for improvement. J Ambul Care Manag.

[61] Bachmann C, Roschlaub S, Harendza S, Keim R, Scherer M (2017). Medical students' communication skills in clinical education: results from a cohort study. Patient Educ Couns.

[62] Zandbelt LC, Smets EM, Oort FJ, Godfried MH, de Haes HCJM (2004). Satisfaction with the outpatient encounter: a comparison of patients' and physicians' views. J Gen Intern Med.

[63] Rashid A, Forman W, Jagger C, Mann R (1989). Consultation in general practice: a comparison of patients' and doctors' satisfaction. BMJ.

[64] Bidmon S, Elshiewy O, Terlutter R, Boztug Y (2020). What patients value in physicians: analyzing drivers of patient satisfaction using physician-rating website data. J Med Internet Res.

[65] Cömert A, Zill JM, Christalle E, Dirmaier J, Härter M, Scholl I (2016). Assessing communication skills of medical students in objective structured clinical examinations (OSCE) -- a systematic review of rating scales. PloS One.

[66] Fürstenberg S, Helm T, Prediger S, Kadmon M, Berberat PO, Harendza S (2020). Assessing clinical reasoning in undergraduate medical students during history taking with an empirically derived scale for clinical reasoning indicators. BMC Med Educ.

[67] Spafford MM, Schryer CF, Lingard L, Hrynchak PK (2006). What healthcare students do with what they don't know: the socializing power of 'uncertainty' in the case presentation. Commun Med.

[68] Lingard L, Garwood K, Schryer CF, Spafford MM (2003). A certain art of uncertainty: case presentation and the development of professional identity. Soc Sci Med.

