# Supplementary material for: Development and pilot test of ComCare – a questionnaire for quick assessment of communicative and social competences in medical students after interviews with simulated patients
Source: GMS J Med Educ. 2021 Mar 15;38(3):Doc68. doi: 10.3205/zma001464 (PMC7994874; doi:10.3205/zma001464)
Supplement: ComCareP [file JME-38-3-68-s-001.pdf]

Attachment 1: ComCareP

**Please read each item and decide to what extent you agree or disagree.**	**not at all**	**hardly**	**partly**	**mostly**	**entirely**	**not assessable**
1. The physician used language I could understand.	○	○	○	○	○	○
2. The physician listened to me attentively.	○	○	○	○	○	○
3. The physician showed sincere interest in me as a human being.	○	○	○	○	○	○
4. The physician responded to my questions and needs satisfactorily.	○	○	○	○	○	○
5. The physician was caring and showed compassion.	○	○	○	○	○	○
6. The physician explained the next diagnostic or treatment steps to me in a comprehensible way.	○	○	○	○	○	○
7. The physician behaved in a way that made me feel comfortable around him/her.	○	○	○	○	○	○
8. I am satisfied with the consultation.	○	○	○	○	○	○

Attachment 1 to: Gärtner J, Prediger S, Harendza S. *Development and pilot test of ComCare – a questionnaire for quick assessment of communicative and social competences in medical students after interviews with simulated patients.* GMS J Med Educ. 2021;38(3):Doc68. DOI: 10.3205/zma001464
