# Supplementary material for: Development and pilot test of ComCare – a questionnaire for quick assessment of communicative and social competences in medical students after interviews with simulated patients
Source: GMS J Med Educ. 2021 Mar 15;38(3):Doc68. doi: 10.3205/zma001464 (PMC7994874; doi:10.3205/zma001464)
Supplement: ComCareD [file JME-38-3-68-s-002.pdf]

Attachment 2: ComCareD

**Please remember the four conversations you just completed. Then please read each item and decide to what extent you agree or disagree.**	**not at all**	**hardly**	**partly**	**mostly**	**entirely**	**not assessable**
1. I used language the patients could understand.	○	○	○	○	○	○
2. I listened to the patients attentively.	○	○	○	○	○	○
3. I showed sincere interest in my patients as human beings.	○	○	○	○	○	○
4. I responded to the patients' questions and needs satisfactorily.	○	○	○	○	○	○
5. I was caring and showed compassion.	○	○	○	○	○	○
6. I explained the next diagnostic or treatment steps in a way that was comprehensible for the patients.	○	○	○	○	○	○
7. I behaved in a way that made my patients feel comfortable around me.	○	○	○	○	○	○
8. I am satisfied with my performance in the consultations.	○	○	○	○	○	○

Attachment 2 to: Gärtner J, Prediger S, Harendza S. *Development and pilot test of ComCare – a questionnaire for quick assessment of communicative and social competences in medical students after interviews with simulated patients.* GMS J Med Educ. 2021;38(3):Doc68. DOI: 10.3205/zma001464
